# Identification and analysis of novel endometriosis biomarkers *via* integrative bioinformatics

**DOI:** 10.3389/fendo.2022.942368

**Published:** 2022-10-20

**Authors:** Sung-Jin Bae, Yunju Jo, Min Kyoung Cho, Jung-Sook Jin, Jin-Young Kim, Jaewon Shim, Yun Hak Kim, Jang-Kyung Park, Dongryeol Ryu, Hyun Joo Lee, Jongkil Joo, Ki-Tae Ha

**Affiliations:** ^1^ Department of Molecular Biology and Immunology, Kosin University College of Medicine, Busan, South Korea; ^2^ Department of Molecular Cell Biology, Sungkyunkwan University School of Medicine, Suwon, South Korea; ^3^ Korean Medical Research Center for Healthy Aging, Pusan National University, Yangsan, South Korea; ^4^ Department of Korean Medical Science, School of Korean Medicine, Pusan National University, Yangsan, South Korea; ^5^ Department of Biochemistry, Kosin University College of Medicine, Busan, South Korea; ^6^ Department of Anatomy, School of Medicine, Pusan National University, Yangsan, South Korea; ^7^ Department of Biomedical Informatics, School of Medicine, Pusan National University, Yangsan, South Korea; ^8^ Department of Korean Medicine Obstetrics and Gynecology, Pusan National University Korean Medicine Hospital, Yangsan, South Korea; ^9^ Department of Obstetrics and Gynecology, Pusan National University Hospital, Busan, South Korea

**Keywords:** endometriosis, LY96, PDLIM3, PTGIS, TLR4/NF-κB, Wnt/frizzled, estrogen receptor

## Abstract

Endometriosis is a gynecological disease prevalent in women of reproductive age, and it is characterized by the ectopic presence and growth of the eutopic endometrium. The pathophysiology and diagnostic biomarkers of endometriosis have not yet been comprehensively determined. To discover molecular markers and pathways underlying the pathogenesis of endometriosis, we identified differentially expressed genes (DEGs) in three Gene Expression Omnibus microarray datasets (GSE11691, GSE23339, and GSE7305) and performed gene set enrichment analysis (GSEA) and protein–protein interaction (PPI) network analyses. We also validated the identified genes *via* immunohistochemical analysis of tissues obtained from patients with endometriosis or healthy volunteers. A total of 118 DEGs (79 upregulated and 39 downregulated) were detected in each dataset with a lower (fold change) FC cutoff (log2|FC| > 1), and 17 DEGs (11 upregulated and six downregulated) with a higher FC cutoff (log2|FC| > 2). KEGG and GO functional analyses revealed enrichment of signaling pathways associated with inflammation, complement activation, cell adhesion, and extracellular matrix in endometriotic tissues. Upregulation of seven genes (*C7*, *CFH*, *FZD7*, *LY96*, *PDLIM3*, *PTGIS*, and *WISP2*) out of 17 was validated *via* comparison with external gene sets, and protein expression of four genes (*LY96*, *PDLIM3*, *PTGIS*, and *WISP2*) was further analyzed by immunohistochemistry and western blot analysis. Based on these results, we suggest that TLR4/NF-κB and Wnt/frizzled signaling pathways, as well as estrogen receptors, regulate the progression of endometriosis. These pathways may be therapeutic and diagnostic targets for endometriosis.

## Introduction

Endometriosis is a common gynecological disorder in which endometrial tissue grows outside the uterus (1). Endometriosis affects around 10% of women of reproductive age and often causes dysmenorrhea, chronic pelvic pain, and infertility (1, 2). Despite the widespread acceptance of the retrograde menstruation theory proposed by Sampson in 1927, the pathogenesis of endometriosis remains poorly understood (3). Thus, various other factors, including genetic, epigenetic, stem cell, inflammatory, angiogenic, and immunological factors, should be considered to better understand the complex pathophysiology of endometriosis (3, 4). Gynecological surgery is the major therapeutic option for endometriosis treatment. Oral contraceptives, progestins, nonsteroidal anti-inflammatory drugs, and gonadotropin-releasing hormone agonists are alternative treatment options (2, 5). However, the efficacy of these treatment strategies, whether surgical or non-surgical, is still limited due to the high recurrence rate of the disease.

Despite its high prevalence, the diagnosis of endometriosis is often delayed as it has no symptoms distinct from those of ordinary menstrual cramps (6, 7). Histopathological examination by invasive laparoscopy or surgery is the gold standard for diagnosis of endometriosis (2). The identification of diagnostic biomarkers is thus urgently required to improve the diagnosis and treatment of patients with endometriosis. Previous reviews on this topic have focused on identification of potential biomarker candidates from specimens such as peritoneal fluid, blood, urine, and endometrial biopsies (6–9), highlighting several factors as noninvasive diagnostic biomarkers, including growth factors, hormones, cytokines, complements, glycoproteins, and antibodies. However, these biomarkers are merely used to supplement diagnosis of endometriosis, as none of them has demonstrated sufficient sensitivity and specificity (10).

Integration of different types of omics data is routinely utilized to discover and validate novel disease biomarkers (11, 12). Potential diagnostic biomarkers and therapeutic targets of endometriosis have been proposed in such integrative bioinformatics studies based on the identification of differentially expressed genes (DEGs) (13–16). However, identification of common biomarkers that are consistently detected in all datasets is difficult due to the heterogeneity among independent datasets. Here, three microarray datasets (GSE11691, GSE23339, and GSE7305), which include gene expression data from normal endometrial and endometriosis tissues, were obtained from the Gene Expression Omnibus (GEO) database. Non-biased bioinformatics analyses, including identification of DEGs, gene set enrichment analysis (GSEA), and protein–protein interaction (PPI) network analysis, were conducted, and the findings were further validated by analyzing immunohistochemistry (IHC) and western blot analysis of tissue specimens obtained from patients with endometriosis or healthy volunteers. Based on the obtained results, we propose six biomarkers as potential targets for the diagnosis and treatment of endometriosis.

## Materials and methods

### Data collection

The datasets for analysis in this study were chosen according to the inclusion and exclusion criteria summarized in **Supplementary Figure 1**. Briefly, the gene sets were obtained by searching the Gene Expression Omnibus (GEO; https://www.ncbi.nlm.nih.gov/geo) database and filtered by three different criteria, including characteristics of data, experiment, and sample. Among six datasets filtered by three criteria, GSE11691, GSE23339, and GSE7305 were applied for DEG and GSEA analysis. Whereas GSE135485 and GSE25628 were used as validation datasets due to their imbalanced and small sample sizes, GSE6364 was filtered out as it contained the data from normal endometrium but not endometriosis. The gene expression data used in this study (GSE11691, GSE23339, GSE7305, GSE135485, and GSE25628) were downloaded from the GEO database, and a total of 128 samples were collected. GSE11691 using the GPL96 platform includes data obtained from nine endometriosis and normal uterine endometrium samples, respectively (17). GSE23339 includes data derived from 10 endometriosis and nine normal uterine endometrium samples using the GPL6102 platform I (18). GSE7305 using the GPL570 platform includes data collected from 10 endometriosis and normal uterine endometrium samples, respectively (19). GSE135485 includes data collected from 54 endometriosis and four normal uterine endometrium samples using the GPL21290 platform. GSE25628 includes data from seven endometriosis and six normal uterine endometrium samples using the GPL571 platform (20). Detailed information on the datasets is summarized in **Table 1**.

**Table 1:** Details of endometriosis GEO data used in this study.

GEO	Author (year)	Platform	Method	Samples	Normal	Endometriosis	PMID
GSE11691	Hull et al (2008)	GPL96	Microarray	Endometrium	9	9	18688027
GSE23339	Hawkins et al (2011)	GPL6102	Microarray	Endometrium	9	10	21436257
GSE7305	Hever et al. (2007)	GPL570	Microarray	Endometrium	10	10	17640886
GSE135485	Yana et al. (2019)	GPL21290	NGS	Endometrium	4	53	–
GSE25628	Crispi et al. (2013)	GPL571	Microarray	Endometrium	6	7	23460397

### Data processing and identification of DEGs

Transcriptome analysis was conducted using R (version 4.1.1) *via* RStudio (Desktop version, 1.4.1717). The three datasets selected for DEG identification (GSE11691, GSE23339, and GSE7305) were downloaded from the GEO database of the National Center for Biotechnology Information using the GEOquery R package (17–19, 21). Multiple probes related to the same gene were reduced to one, and summarized as median values for further analysis. Since gene expression profiles differed between samples included in GSE11691, quantile normalization was applied using the preprocessCore R package (https://github.com/bmbolstad/preprocessCore). DEGs were defined as genes with adjusted *p*-values and Log2|FoldChange| (Log|FC|) less and greater than 0.05 and 1, respectively. We adjusted *p*-value to correct the false positive error caused by the multiple tests and calculated it by the Benjamini & Hochberg method (22), which is one of the popular tools to minimize the false discovery rate. The cutoff criteria that we applied was 0.1. For analysis of the pathway and PPI of DEGs, we adopted Log|FC| > 1 and used Log|FC| > 2 for heatmap and network analysis.

### Pathway enrichment analysis

Analysis of Kyoto Encyclopedia of Genes and Genomes (KEGG) and Gene Ontology (GO) biological processes of DEGs were performed using the JEPETTO plugin (version 1.3.1) of Cytoscape (version 3.8.2). Visualization was performed by constructing a scatter plot with XD-score and *q*-value as axes. *q*-value < 0.25 was used as the cutoff criterion, according to GSEA guidance (https://software.broadinstitute.org/cancer/software/gsea/wiki/index.php/FAQ).

### PPI network analysis

The interactions of proteins encoded by the DEGs were identified using the STRING plugin (version 1.7.0) of Cytoscape by using “*Homo sapiens*” as search keyword, and a confidence score cutoff higher than 0.4. GeneMANIA plugin (version 3.5.2) was used to identify physical interactions. Visualization of the network was performed using Cytoscape.

### Heatmap construction and network analysis of DEGs

A heatmap of the top 17 DEGs identified in each dataset was generated using Morpheus, a versatile matrix visualization and analysis software from the Broad Institute at the Massachusetts Institute of Technology (https://software.broadinstitute.org/morpheus/).

Network analysis was performed as previously described (23). Briefly, the pattern of co-expression between the normal and endometriosis groups was visualized based on Spearman’s correlation. For this purpose, dplyr, stringr, ggpubr, ggplot2, igraph, ggraph, corrr, corrplot, tidyverse, and reshape2 R packages were applied.

### GSEA

GSEA of each gene expression dataset was performed using the GSEA software (version 4.1.0) from the Broad Institute at the Massachusetts Institute of Technology (https://www.gsea-msigdb.org/gsea/index.jsp). The absolute value of the normalized enrichment score (NES), the enrichment score for the gene set after it had been normalized across analyzed gene sets, was set to > 1.5 as the cutoff criteria. The false discovery rate (FDR) *q*-value, which represents the estimated probability that the normalized enrichment score constitutes a false-positive finding, was set to < 0.02. GO network analyses were performed on each GSEA by utilizing Cytoscape to visualize enrichment maps.

### Clinical sample collection

Laparoscopic surgeries were performed at the Department of Obstetrics and Gynecology of Pusan National University Hospital (Busan, Korea) between June 2019 and December 2020. The surgical process was performed during the early follicular phase to rule out the potential early pregnancy and the possibility of the ovarian cyst being functional (24, 25). To obtain normal uterine endometrial tissue as the control group, we gathered the patients with male-factor infertility with normal gynecologic anatomy who underwent endometrial scratch prior to proceeding to programmed or natural embryo-transfer cycle, and the patients with hormone-independent ovarian cysts such as mature teratoma or cystadenoma who underwent laparoscopy and presented no endometriotic lesion in the pelvic cavity. The exclusion criteria were irregular menstrual periods, the presence of endocrine disorders such as hyperprolactinemia or thyroid dysfunction, and medication history with dysmenorrhea management such as GnRH analogs, oral contraceptives, or progestins during the past three months to the recruitment. Eligible patients were further examined and proven morphologically free from possible asymptomatic endometriosis and other hormone-dependent gynecological pathologies, such as uterine adenomyosis and leiomyoma, by undergoing imaging studies including gynecological ultrasonography, abdominal computed tomography scans and/or pelvic magnetic resonance imaging. All specimens from the control group were further histologically assessed and confirmed as normal endometrial tissue by pathologists. Endometriosis was pathologically diagnosed in tissue specimens derived from 32 patients. Endometriosis was classified into stages I-IV according to the revised American Society for Reproductive Medicine (r-ASRM) classification system. The average stage detected in the samples was approximately III. The average age of the patients was 33.33 ± 7.53 years, whereas the average BMI was 21.51 ± 3.52. Samples of normal endometrial tissue were obtained from ten healthy volunteers who have not been diagnosed with endometriosis. The average age of the volunteers was 30.3 ± 4.9 years, whereas the average BMI was 22.06 ± 2.06. There was no statistically significant difference between the average age and BMI values of the two groups (with *p*-values of 0.16 and 0.56). Tissue specimens were deposited in the Biobank of the Pusan National University Hospital. Detailed information on the patient and normal volunteer is summarized in **Supplementary Table 1**.

This study was approved by the Institutional Review Board (IRB) of Pusan National University Hospital (2104-009-101). All patients signed informed consents for the study protocol. All procedures were conducted in accordance with IRB guidelines.

### IHC analysis

Fresh tissues collected by the laparoscopic surgeries were rinsed with normal saline to remove blood and impurities, and rapidly frozen and stored in the Biobank of Pusan National University Hospital. The donated specimens were fixed with 4% formaldehyde solution, and processed to obtain paraffin embedded tissue blocks. Five–μm thick sections of tissue blocks were deparaffinized by soaking in xylene and gradient ethanol solution. The sections were then incubated with primary antibodies, including anti-lymphocyte antigen 96 (LY96; 1:200; ab22048, Abcam), anti-PDZ And LIM Domain 3 (PDLIM3; 1:200; HPA004749, Atlas Antibodies, Bromma, Sweden), anti-prostaglandin I2 synthase (PTGIS; 1:200; ab23668, Abcam), and anti-WNT1-inducible-signaling pathway protein 2 (WISP2; 1:200; ab28317, Abcam) at 4°C overnight. The sections were then incubated with Dako REAL EnVision Detection System (K5007; Dako, Jena, Germany) for 1 h. After rinsing with phosphate-buffered saline (PBS), immunostaining was visualized using DAB+ chromogen buffer (K5007, Dako). The slides were counterstained with hematoxylin solution, and representative images were taken using an optical microscope (Axio Scope A1; Carl Zeiss, Oberkochen, Germany). Histopathological scoring was performed through examination under a light microscope by a pathologist. Staining intensity was classified as follows: 0, negative immunostaining; 1, weak expression level; 2, moderate expression level; 3, strong expression level; and 4, very strong expression level.

### Western blot analysis

Total proteins were extracted from frozen tissue specimens using protein lysis buffer containing 10 mM HEPES pH 7.45, 150 mM sodium chloride, 1% (*w*/*v*) NP−40, 5 mM sodium pyrophosphate, 5 mM sodium fluoride, and 2 mM sodium vanadate with a protease inhibitor cocktail (Roche Applied Science, Penzburg, Germany). 30 μg of protein lysates was electrophoresed by sodium dodecyl sulfate−polyacrylamide gel and transferred onto nitrocellulose membranes (0.45 μm; ThermoFisher Scientific, Waltham, MA). The membranes were blocked with 5% (*w*/*v*) non-fat dry milk and incubated with primary antibodies against target proteins, including LY96, PDLIM3, PTGIS, and WISP2 at 4°C overnight. The membranes were washed and incubated with proper secondary antibodies conjugated with horseradish peroxidase. The bands of interesting proteins were detected with the enhanced chemiluminescence Plus kit (ThermoFisher Scientific) using ImageQuant LAS 4000 imaging system (GE healthcare, Chicago, IL).

### Identification of potential druggable genes

The Drug-Gene Interaction Database (DGIdb, http://www.dgidb.org) is an online database that facilitates interpretation of the results of genome-wide studies, and generation of hypotheses in the context of druggable genome (26). DGIbd was used to identify potentially druggable genes from an input list of genes including the statistically significant DEGs.

### Statistical analysis

To evaluate the statistical difference between the two groups, Student’s *t*-test was performed using GraphPad Prism (version 5.01; GraphPad Software, San Diego, CA, USA). Statistical significance was set at *p* < 0.05.

## Results

### Identification of DEGs

**Figures 1:**
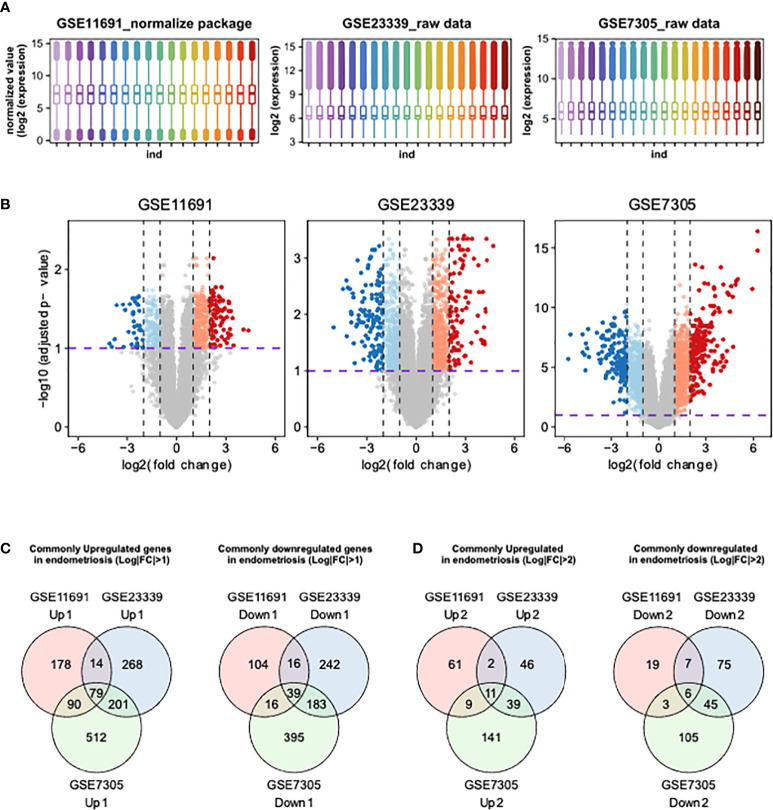
Identification of DEGs using integrative bioinformatics analysis. **(A)** The distribution of transcriptome obtained from GSE11691, GSE23339, and GSE7305 were shown. **(B)** The volcano plots show the DEGs from GSE11691, GSE23339, and GSE7305. Red indicates relative upregulated genes with *p*-value < 0.05 and Log2|FC| > 2, orange indicates relative upregulated genes with *p*-value < 0.05 and 1 < Log2|FC| ≤ 2, blue indicates relative downregulated genes with *p* < 0.05 and Log2|FC| > 2, and light cyan indicates relative downregulated genes with *p* < 0.05 and 1 < Log2|FC| ≤ 2. **(C, D)** Venn diagrams of upregulated and downregulated genes DEGs from three indicated datasets. The cutoff values of Log2|FC| were set to 1 **(C)** and 2 **(D)**.

We first determined the distributions of gene expression levels in three human endometrial transcriptome datasets, GSE11691, GSE23339, and GSE7305 (17–19). The transcriptome profiles of GSE11691 showed the highest level of variation among the three transcriptomes. Thus, we conducted quantile normalization to avoid artifacts in subsequent analyses. The gene expression data included in GSE23339 and GSE7305 datasets were converted to Log2 scale (**Figure 1A**), and DEGs were then identified using these three datasets. As highlighted in the colored volcano plots (**Figure 1B**), a total of 536 (361 upregulated and 175 downregulated), 1,042 (562 upregulated and 480 downregulated), and 1,515 (882 upregulated and 633 downregulated) DEGs were identified in GSE11691, GSE23339, and GSE7305, respectively (**Figure 1C**). Moreover, integration of DEGs shared across the datasets revealed 118 (79 upregulated and 39 downregulated; **Figure 1C**) and 17 (11 upregulated and six downregulated; **Figure 1D**) common DEGs with a lower (fold change) FC cutoff (log2|FC| > 1) and a higher FC cutoff (log2|FC| > 2), respectively (**Table 2**).

**Table 2:** The list of identified DEGs in three endometriosis datasets, GSE11691, GSE23339, and GSE7305.

DEGs	Gene names
Up-regulated (27)	*AEBP1, AGTR1, BGN, C3, C3AR1, C7, CCL2, CD14, CD163, CFH, CHL1, COL11A1, COL14A1, COLEC12, CPE, CPVL, CSTA, CXCL12, DKK3, DPYD, DPYSL3, DSE, ELMO1, EVI2B, FABP4, FAM129A, FCGR2A, FCHSD2, FGL2, FMO1, FMO2, FMOD, FN1, FRZB, FZD7, GAS1, HEG1, HLA_DPA1, HLA_DPB1, HLA_DQA1, HLA_DRA, IGJ, IL4R, ITM2A, KCTD12, LAMA4, LHFP, LTBP2, LY96, LYZ, MEIS2, MMP23A, MN1, MNDA, MS4A4A, MS4A6A, NUAK1, OLFML1, PDE1A, PDGFRL, PDLIM3, PLSCR4, PLXDC2, PRELP, PTGER4, PTGIS, RNASE1, RNASE6, ROBO3, SGCE, SULF1, TAGLN, TCEAL2, THBS2, TNFSF14, TYROBP, VCAM1, VSIG4, WISP2*
Down-regulated (28)	*ACSL5, AGR2, AP1M2, BTBD3, CDS1, CLDN10, CLDN3, CNDP2, DEFB1, EDN3, ELF3, GALNT4, GRAMD1C, GRHL2, HMGCR, HOOK1, HOXB6, HPN, HSD17B2, IL20RA, IRF6, ITGB8, KIAA1324, MME, OSR2, PAPSS1, PEMT, PERP, PLS1, PPM1H, PRSS16, PRSS8, PTPN3, RAB25, SH3YL1, SLC3A1, SPINT2, TPD52L1, TSPAN1*

Red and blue characters indicate genes enriched in endometriosis and normal endometrial tissues, respectively (log2|FC|>2.0).

### Pathway enrichment analysis

**Figure 2:**
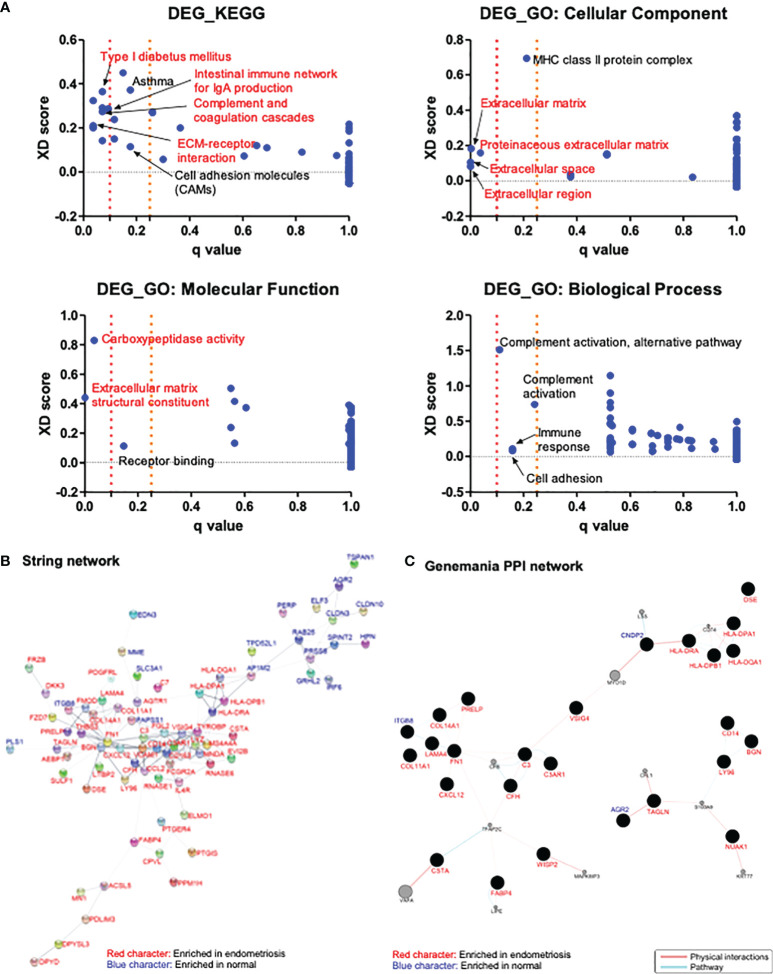
Analysis of the enriched pathways and interaction networks of DEGs in endometriosis. **(A)** KEGG and GO pathway analysis of DEGs from GSE11691, GSE23339, and GSE7305 datasets with Log2|FC| > 1 were performed with JEPETTO plugin of Cytoscape, and scatter plot were constructed with *q*-value and XD-score as *x*- and y-axis, respectively. Red character indicates the pathways with *q*-value < 0.1 and black character indicates the pathways with 0.1 < *q*-value < 0.25. **(B, C)** Protein–protein interaction network of DEGs from three datasets with Log2|FC| > 1 were analyzed in Cytoscape with plugins of STRING **(B)** and GeneMANIA **(C)**. Red character indicates genes enriched in endometriosis and blue character indicates genes enriched in normal.

To elucidate the pathways and molecular functions related to the 118 common DEGs identified above, KEGG and GO enrichment analyses were conducted. The analysis results revealed the involvement of the identified DEGs in the extracellular matrix, cell adhesion, complement activation, immune response, and inflammation processes (**Figure 2A**).

### PPI network analysis

Interactions between proteins encoded by 118 DEGs identified above were analyzed using the STRING and GeneMANIA plugins of Cytoscape. STRING analysis resulted in a network comprising 118 nodes (genes) and 197 edges (interactions). A total of 37 nodes did not have any edges, and four nodes had only few edges. The rest of the network, including 77 genes and 195 interactions is shown in **Figure 2B**. The genes *AP1M2*, *BGN, C3, C3AR1, CCL2, CD14, CD163, COL14A1, FCGR2A, FGL2, FN1, LYZ, MS4A4A, RNASE6, TYROBP*, and *VCAM1* had relatively higher number of interactions (over four edges). GeneMANIA analysis resulted in a network consisting of 138 nodes and 72 edges. Among these nodes, 77 genes did not have any physical or pathway interactions, and 24 genes had merely simple interactions. The main interaction network harboring 37 nodes and 57 edges is shown in **Figure 2C**. The genes showing relatively higher number of interactions (over three edges) in the network were identified to be *C3, CFH, CNDP2, COL11A1, COL14A1, FABP4, FN1, HLA-DPA1, HLA-DPB1, HLA-DQA1, HLA-DRA, ITGB8, LAMA4, LY96, and TAGLN*. STRING analysis revealed *CCL2* as significant (with over three edges), based on 15 common DEGs with a cutoff value of Log2|FC| > 2. On the other hand, GeneMANIA revealed that three proteins encoded by *AGR2, FZD7*, and *LY96* were in physical or pathway contact with each other (**Supplementary Figure 2**).

### Network analysis of DEGs

**Figure 3:**
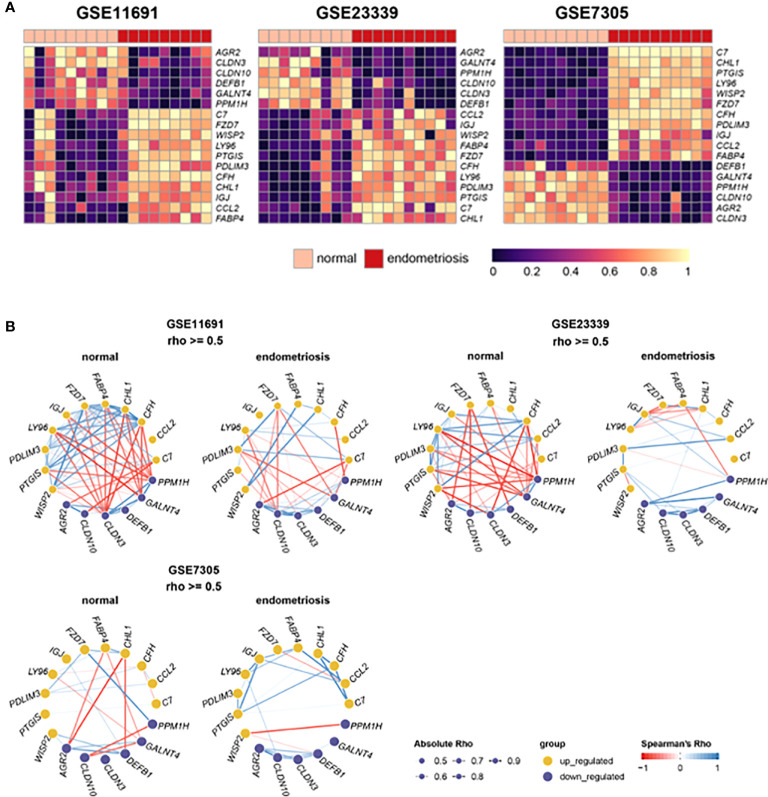
Expression and correlation of 22 DEGs with Log2|FC| > 2. **(A)** Heat maps show the expression levels of 22 DEGs in datasets, GSE11691, GSE23339, and GSE7305. **(B)** Gene networks display correlations of 22 DEGs in the normal and endometrial tissues of each dataset. The depth of edges indicates absolute Rho ranging from 0.5 to 1. The color of edges indicates co-expression ranging from -1 (red) to 1 (blue) by Spearman’s Rho.

The heatmaps (**Figure 3A**) depicted the expression levels of the 17 frequent DEGs with *p*-values less than 0.05 and Log2|FC| more than 2, clearly illustrating the differential expression patterns of those selected genes in endometriosis compared to normal control across three independent datasets. In addition, gene networks showing the co-expression patterns of the selected genes were constructed based on Spearman’s correlation. The 17 DEGs were highly associated with one another in three independent datasets from normal tissues, and thereby generated a massive co-expression gene network (**Figure 3B**). On the other hand, the number of correlations, shown as edges, was drastically reduced in ectopic lesions of endometriosis patients compared to normal sites. Interestingly, these patterns of gene networks with decreased correlations under endometriosis were comparable across the three independent datasets. Hence, the decrease in correlations between these 17 DEGs may contribute to the pathophysiology of endometriosis.

### Identification of functional pathways by GSEA

**Figure 4:**
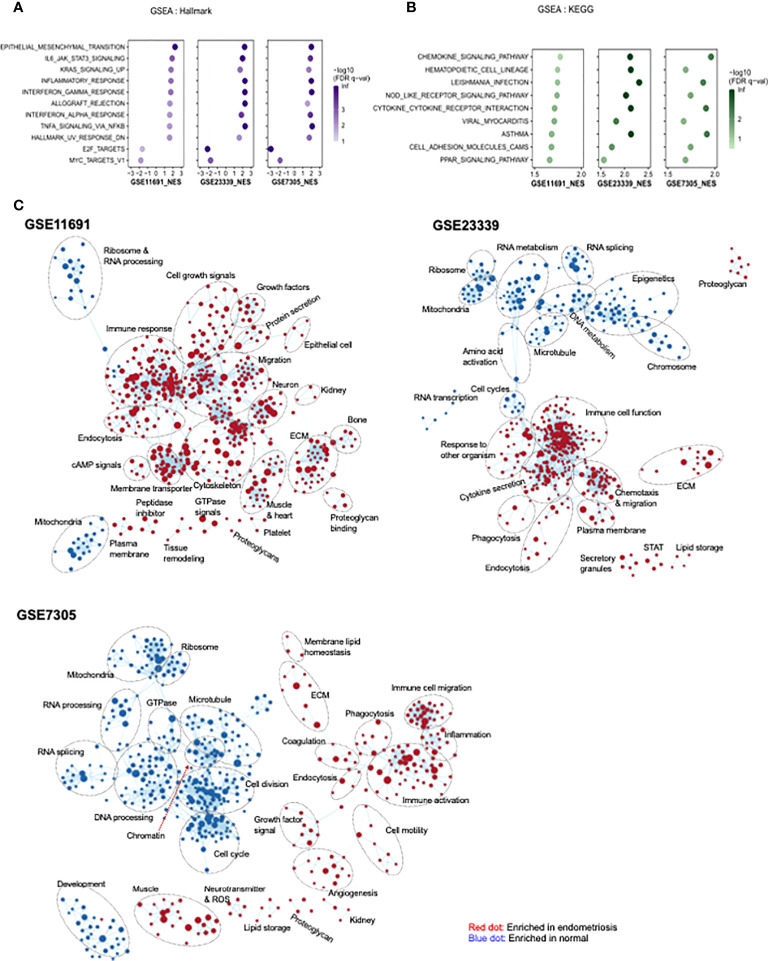
Identification of pathway networks by GSEA. **(A, B)** Common pathway categories identified by GSEA with gene set database of Hallmark **(A)** and KEGG **(B)** are presented. **(C)** GSEA was performed to obtain enriched GO-terms and visualized using Enrichment Map plugin of Cytoscape. The size of each node indicates the size of gene set. Red and blue indicate the node enriched in endometriosis and normal tissue, respectively.

GSEA was carried out to identify functional pathways in endometriosis that are shared across the three datasets analyzed (GSE11691, GSE23339, and GSE7305). A total of 11 common pathways were identified in the hallmark analysis, including 9 upregulated and 2 downregulated pathways (**Figure 4A**). Inflammation-related pathways such as IL6-JAK/STAT3 signaling, inflammatory response, interferon α response, interferon γ response, and TNFα signaling *via* NF-κB, were found to be significantly upregulated in patients with endometriosis. In addition, nine common pathways were found to be upregulated in KEGG analysis (**Figure 4B**). Further, inflammation-related pathways including chemokine signaling pathway and NOD-like receptor signaling pathway, cytokine-cytokine receptor interaction, viral myocarditis, Leishmania infection, and asthma were found to be upregulated in patients with endometriosis. In GO network analysis, inflammation-related pathways, such as immune response, chemotaxis, and immune cell migration, and cell-cell interaction-related pathways, such as extracellular matrix, proteoglycan, and endocytosis, were commonly enriched for genes of three datasets with endometriosis (**Figure 4C**). Phagocytosis-related pathways were upregulated in endometriosis in two datasets, GSE23339 and GSE7305. The small portions of pathways related to platelets and coagulation were upregulated in endometriosis of the two gene sets, GSE11691 and GSE7305. GO pathways, including mitochondria, ribosomes, and RNA splicing and/or processing, were commonly enriched in three normal datasets. Finally, the GO pathways involved in the cell cycle and microtubules were downregulated in two datasets, GSE23339 and GSE7305.

### Protein expression of identified DEGs in endometriosis lesions

To further evaluate the reliability of the results from integrative analysis of GSE11691, GSE23339, and GSE7305, the expression levels of 17 common DEGs in two external datasets, GSE135485 and GSE25628, were determined. The expression levels of 10 genes in GSE135485, including *C7, CFH, CHL1, CLDN3, FZD7, IGJ, LY96, PDLIM3, PTGIS*, and *WISP2* were correlated with those in the validation sets (**Supplementary Table 2**). However, the expression levels of other genes did not correlate with those in the integrated data. In the GSE25628 dataset, expression levels of 11 genes, including *AGR2*, *C7, CFH, FABP4, FZD7, GALNT4, LY96, PDLIM3, PPM1H*, *PTGIS*, and *WISP2* were in line with integrative analysis results (**Supplementary Table 3**). According to results from two external datasets, GSE135485 and GSE25628, the expression levels of seven genes, including *C7*, *CFH*, *FZD7*, *LY96*, *PDLIM3*, *PTGIS*, and *WISP2* were correlated with training sets listed in **Table 2**.

**Figure 5:**
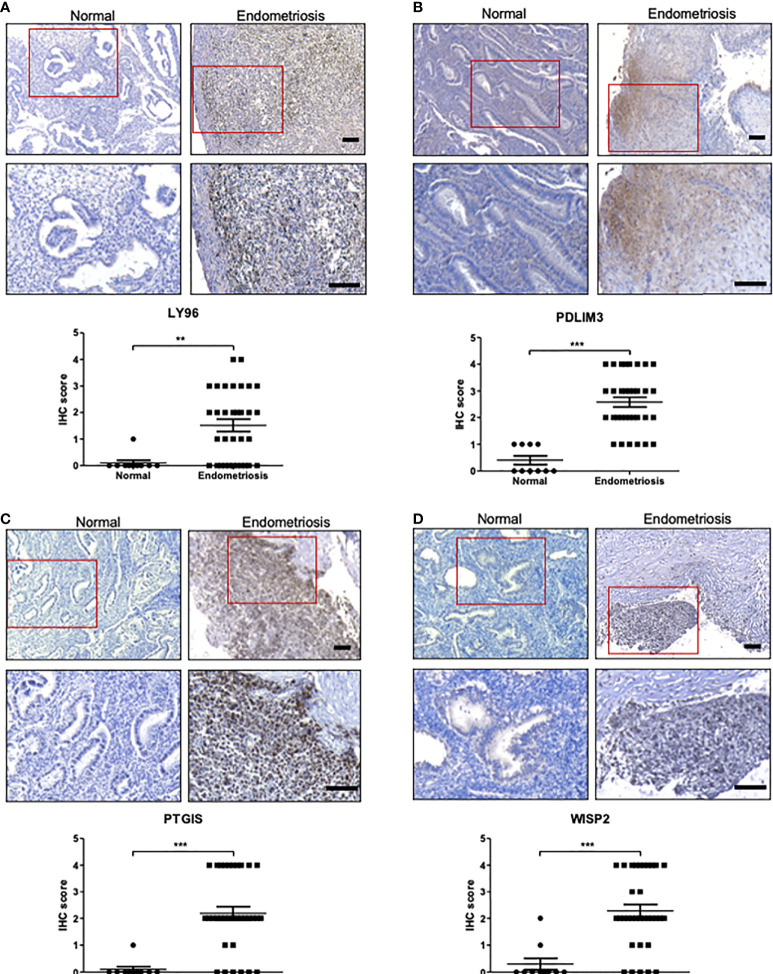
The protein expression of LY96, PDLIM3, PTGIS, and WISP2 in endometriosis tissue. **(A–D)** The protein expression of **(A)** LY96, **(B)** PDLIM3, **(C)** PTGIS, and **(D)** WISP2 was measured by IHC analysis of endometrial tissues from healthy volunteers or endometriosis patients. The representative IHC images are shown in upper panel (bar = 100 μm). The graphs in lower panel represent IHC score. **p < 0.01 and ***p < 0.001.

The roles of *C7*, *CFH*, and *FZD7* in endometriosis have been reported previously (8, 29). These studies revealed higher expression of C7, CFH, and FZD7 proteins in tissues with endometriosis compared to normal endometrium by IHC analysis. High expression of *PDLIM3* in endometriosis has also been reported in several previous bioinformatics studies with little supportive experimental evidence (15, 30, 31). Thus, we conducted IHC and western blot analysis on tissues from normal endometrium and endometriosis foci to further evaluate the expression of proteins encoded by the identified genes, including *LY96*, *PDLIM3*, *PTGIS*, and *WISP2*. None of these genes have yet been evaluated in terms of their expression profiles in endometriosis tissue. Quantitative assessments of histological images clearly demonstrated higher expression levels of LY96, PDLIM3, PTGIS, and WISP2, proteins in tissues from patients with endometriosis, in line with integrative analysis results (**Figure 5**). According to western blot analysis, the protein levels of LY96, PDLIM3, and PTGIS were higher in the tissues of endometriosis patients. However, the expression of WISP2 was not correlated with the IHC analysis (**Supplementary Figure 3**).

### Identification of potent druggable genes

To further identify relevant drug-gene interactions and potential druggable target genes, we utilized the DGIdb by applying *C7*, *CFH*, *FZD7*, *LY96*, *PDLIM3*, and *PTGIS* as queries. Three genes, *FZD7*, *LY96*, and *PTGIS*, were found to be associated with drugs vantictumab, eritoran tetrasodium, and phenylbutazone, respectively (**Table 3**). *CFH* was predicted to be a target gene for three antibody drugs: bevacizumab, eculizumab, and ranibizumab. No potential gene-drug interactions were identified for *C7* and *PDLIM3*.

**Table 3:** Potent drugs identified in DGIdb corresponding to consistently upregulated six DEGs in endometriosis.

Gene	Drug	Approved	Interaction score	Interaction types	Drug class	PMID
*C7*	–	–	–	–	–	–
*CFH*	ECULIZUMAB	Yes	14.14	na	Antibody	19854549, 27799617
	BEVACIZUMAB	Yes	7.53	na	Antibody	26439641
	RANIBIZUMAB	Yes	58.35	na	Antibody	21558292, 22840423
*FZD7*	VANTICTUMAB	No (Clinical trial)	27.28	Antagonist	Antibody	22753465
*LY96*	ERITORAN TETRASODIUM	No (Clinical trial)	31.83	Antagonist	–	23512062
*PDLIM3*	–	–	–	–	–	–
*PTGIS*	PHENYLBUTAZONE	Yes (For animal use)	37.13	Inhibitor	Non-steroidal anti-inflammatory agent	3917545, 6434940

## Discussion

Endometriosis is a chronic inflammatory disease. The infiltration of immune cells and secretion of inflammatory mediators in the peritoneal microenvironment cause symptoms and signs observed in endometriosis patients (32). Endometriosis is characterized by inflammation and subsequent fibrosis, which eventually lead to pelvic discomfort, bowel and urinary problems, and infertility (32, 33). In contrast to normal endometrium, accumulated hemorrhage and tissue injury are key steps for initiating inflammation in the endometrial lesion and peritoneal cavity (33, 34). Hormonal alterations, particularly cyclic estrogen fluctuations, also contribute to an inflammatory imbalance in endometriosis (35). In contrast to other inflammatory diseases, increased estradiol production and estrogen receptor β (ERβ) expression in ectopic endometriosis lesions jointly activate nuclear factor-κB (NF-κB), a key inflammatory regulator (36, 37). Thus, the combination of oral contraceptives and non-steroidal anti-inflammatory drugs (NSAIDs) has been advised as the initial medical treatment option (10, 38). However, the clinical efficacy of this combination is suboptimal due to its low potency and side effects.

In this study, we identified six genes that may be utilized as diagnostic and/or therapeutic targets in endometriosis. STRING and GeneMANIA analyses revealed no evidence of any potent protein–protein interaction between these genes. Complement 7 (C7) and complement factor H (CFH), both of which are components of the complement system, have previously been identified as potent indicators of complement activation in endometriosis and endometriosis-associated ovarian cancer (29). C3, another member of the complement system, has been reported to be upregulated in endometriosis (39) and was also identified in our study as a common upregulated gene. Other members of the complement activation pathway, such as complement C3a receptor 1 (C3AR1) and V-set and immunoglobulin domain containing 4 (VSIG4), are also known in endometriosis, and upregulation of complement and coagulation pathways in endometriosis has been well-reported in several studies (15, 29, 39, 40). These studies suggested that autoimmune response in endometriosis is associated with the complement system (29, 39). In addition, the membrane attack complex in the complement system, which is composed of several complement proteins including C5b, C6, C7, C8, and C9, may also cause tissue damage and thereby induce inflammation in endometrial lesions.

Lymphocyte antigen 96 (LY96), also known as myeloid differentiation protein 2 (MD-2), has been reported its expression in the uterine endometrium (28, 41) and is a coreceptor of Toll-like receptor 4 (TLR4) (42). The TLR4 system is crucial for pathogen recognition and the activation of innate immunity. The complex of bacterial lipopolysaccharide (LPS) and LPS-binding protein interacts with cluster of differentiation 14 (CD14), a glycosylphosphatidylinositol-anchored membrane protein, and transfers LPS to LY96, consequently facilitating the dimerization of TLR4 (43). Damage-associated molecular patterns (DAMPs) secreted by most types of damaged tissues also bind to the TLR4 system and activate downstream pro-inflammatory signals similar to pathogen-associated molecular patterns, including bacterial LPS (44). Prostaglandin I2 synthase (PTGIS), also known as prostacyclin synthase (PGIS) or cytochrome P450 isomerase 8A1 (CYP8A1), is an enzyme that converts prostaglandin H2 to prostaglandin I2, and thereby modulates the inflammatory response (45). The role of PTGIS in inflammatory diseases is controversial, as it promotes progression of rheumatoid arthritis yet suppresses progression of pulmonary vascular disease and atherosclerosis (46). Although PTGIS was identified as a transcriptional target gene of NF-κB (47), the regulation of PTGIS mRNA expression does not exactly correlate to those of typical NF-κB targets (48, 49). In the vascular system and uterine endometrium, estrogen receptors work in concert with NF-κB to regulate the activity of prostaglandin-synthesizing enzymes, including COX-2 and PTGIS (50, 51). Furthermore, high levels of prostacyclin and its derivative, 6-keto-prostaglandin F1α, have been detected in the peritoneal fluid of endometriosis patients (52, 53). PTGIS expression was shown to be downregulated in a murine model of implanted endometrium (54); however, proteomics analyses demonstrated upregulated PTGIS expression in human ovarian endometrioma samples (55). In this study, we demonstrated the increased expression of *LY96* and *PTGIS via* integrative transcriptome analysis, and further validated by IHC analysis of human-derived tissues.

Frizzled 7 (FZD7) is a member of the frizzled family and an atypical G protein-coupled receptor for Wnt proteins. FZD7 interacts with Dishevelled (Dvl) and lipoprotein receptor-related proteins (LRPs) in the presence of canonical Wnt signaling, and thereby promotes β-catenin signaling (56). This signaling pathway is closely related to embryonic development, cell proliferation, epithelial-to-mesenchymal transition, and carcinogenesis (57, 58). Wnt signaling is also involved in the production of enzymes related to prostaglandin metabolism in bone and skin, including COX-2 and PTGIS (59, 60). The crosstalk between the Wnt/frizzled and TLR4/NF-κB signaling pathways is well-established in chronic inflammation, development, and tumorigenesis (61, 62). PDZ and LIM domain 3 (PDLIM3) are involved in cytoskeleton assembly, in particular the formation of Z-disks in skeletal muscles (63). Although several microarray and proteome analyses have already revealed high PDLIM3 expression in this regard (15, 30, 31, 55), the exact role of PDLIM3 in endometriosis remains to be elucidated. The expression of Wnt1-inducible signaling pathway protein 2 (WISP2) might be another evidence of the connection between Wnt/frizzled and TLR4/NF-κB signaling pathway. The protein, also known as cellular communication network factor 5 (CCN5), is a secretory protein and a member of the connective tissue growth factor family (64). The expression of WISP2 is induced by Wnt signaling, and restricts cell growth, migration, adhesion, and differentiation, particularly in the vascular system and cancer cells (65–67). Secreted WISP2 is also involved in the activation of the canonical Wnt signaling pathway (68). However, our examination of WISP2 expression was not consistent between IHC and Western blot analysis. the delicate role of the WISP2 in the endometriosis still remains ambiguous and further extensive studies are required.

**Figure 6:**
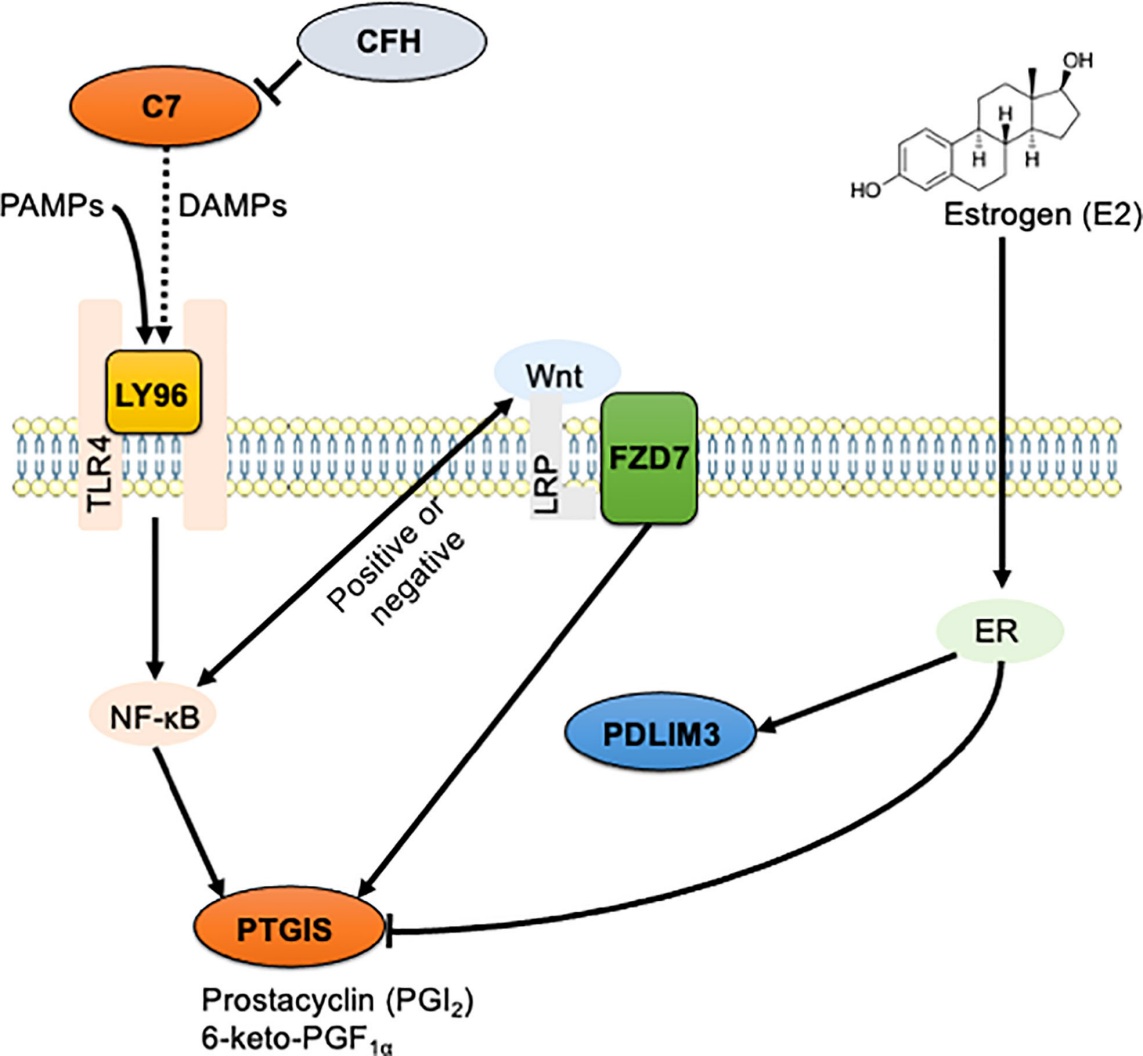
The schematic illustration of the hypothesized signaling network in endometriosis. Signaling interaction between six identified DEGs including *C7*, *CFH*, *FZD7*, *LY96*, *PDLIM3*, and *PTGIS*, and three enriched pathways including TLR4/NF-κB, Wnt/frizzled, and estrogen receptors were hypothesized and schematically illustrated. Solid line indicates direct interaction, and dashed line indicates indirect and/or proposed interaction.

In endometriosis, chronic inflammation is co-regulated by ERβ-related signaling and the classical NF-κB signaling pathway (36, 37). Estrogen stimulation increases the expression of PDLIM3 in human prostate cancer (69). However, even if the expression level of PTGIS does not perfectly match that of the ERβ, it may be negatively influenced by ERβ (70, 71). We thus propose here the existence of signaling interactions between the validated DEGs including *C7, CFH, FZD7, LY96, PDLIM3*, and *PTGIS*, and pathways including TLR4/NF-κB, Wnt/frizzled, and estrogen receptors (**Figure 6**). Further research is needed to elucidate the precise roles of the identified genes in endometriosis.

We also found four druggable genes: *FZD7*, *LY96*, *PTGIS*, and *CFH*. *FZD7* is a direct target of vantictumab, a neutralizing antibody currently being developed as an anticancer agent, particularly for triple-negative breast cancer (72, 73). In addition to vantictumab, an oligopeptide Fz7-21 and a small molecule SRI37892 have been evaluated as a new Fzd7-targeting agent to disrupt the Wnt signaling pathway for inhibiting intestinal stem cell function and cancer progression (74, 75). We suspect that these agents may have been identified for candidate drugs if registration in DGIdb. *LY96* has been identified as a target of eritoran tetrasodium, a TLR4 antagonist drug that has been indicated for the treatment of sepsis in several clinical studies (76). However, eritoran tetrasodium was not successful due to its similar mortality compared to that of placebo (42). Instead of eritoran tetrasodium, we suggest that MD2-IN-1, isofraxidin, and L48H37, studied in the inflammation and cancer research field at the preclinical level, might be applied to treat endometriosis as LY96-targeting agents (77–79). Phenylbutazone is a non-steroidal anti-inflammatory drug (NSAID) that inhibits prostaglandin H synthases (PTGS1 and PTGS2) and PTGIS through peroxide-mediated deactivation (27). Although it has been approved for the treatment of backache and ankylosing spondylitis, phenylbutazone is currently withdrawn from human medicine, as it can cause severe adverse effects such as suppression of white blood cell production and aplastic anemia (80). For CFH-targeting drugs, DGIdb has suggested two VEGF-neutralizing antibodies, including ranibizumab and bevacizumab, and one C5-antagonizing antibody, eculizumab. As CFH is a non-specific off-target for these antibodies, its clinical application may result in significant adverse effects, particularly in patients with a mutated CFH gene (81–85). None of these drugs for targeting identified genes have yet been used to target the identified genes for endometriosis treatment. Additional *in vivo* and clinical studies are needed to determine the efficacy of these drugs in endometriosis treatment.

The datasets should ideally be comparable in terms of sample collection, underlying disease, menstrual cycle, and experiment types for the analysis of the collection of public gene sets. These elements of the datasets used for DEG analysis varied, particularly with regard to the menstrual cycle and underlying diseases such leiomyoma. We tried to select datasets using a filter of three objective criteria despite the restriction of only being able to access the datasets from public databases. In addition, we meticulously gathered normal control samples during the early follicular phase from healthy volunteers who were free of any gynecological diseases. However, there is almost no significant chance of finding genes that follow the menstrual cycle or underlying disease. The results clearly indicate that the genes discovered by DEG analysis may be important for conditions beyond than underlying disease and/or the menstrual cycle.

In conclusion, we identified 118 DEGs (79 upregulated and 39 downregulated) that may be involved in endometriosis pathogenesis. KEGG and GO functional analyses revealed enrichment of inflammation, complement activation, cell adhesion, and extracellular matrix pathways in endometriosis. Six genes, *C7, CFH, FZD7, LY96, PDLIM3*, and *PTGIS* were verified as upregulated DEGs by comparison to external gene sets, IHC and western blot analyses further confirmed the elevated protein expression levels of LY96, PDLIM3, and PTGIS in human endometrial lesions. We further analyzed the involvement of these genes in signaling pathways including TLR4/NF-κB, Wnt/frizzled, and estrogen receptors. We expect that subsequent studies will confirm the genes identified here as essential biomarkers for endometriosis diagnosis and treatment.

## Data availability statement

The original contributions presented in the study are included in the article/**Supplementary Material**. Further inquiries can be directed to the corresponding authors.

## Ethics statement

The studies involving human participants were reviewed and approved by Institutional Review Board (IRB) of Pusan National University Hospital (2104-009-101). The patients/participants provided their written informed consent to participate in this study.

## Author contributions

YJ analyzed informatics data. MC and J-SJ conducted IHC experiments. S-JB, J-YK, and JS analyzed the data. YK and DR validated and visualized the results from the informatics analysis. J-KP, HL, and JJ collected human samples and analyzed the clinical signatures and statistics. S-JB and YJ wrote the draft of this manuscript. S-JB, J-YK, and JS revised the manuscript. JJ and K-TH conceptualized this study and reviewed the manuscript. All authors read and approved the final manuscript.

## Funding

This research was supported by a grant from Kosin University College of Medicine (2021) and by the National Research Foundation of Korea (NRF) grants funded by the Korean government (MIST: NRF-2020R1C1C1003703 to S-JB, NRF-2022R1I1A1A01063460 and NRF-2021R1A5A8029876 to YJ, NRF-2020R1F1A1067185 to JJ, and NRF-2021R1A4A1025662 to S-JB and K-TH).

## Conflict of interest

The authors declare that the research was conducted in the absence of any commercial or financial relationships that could be construed as a potential conflict of interest.

## Publisher’s note

All claims expressed in this article are solely those of the authors and do not necessarily represent those of their affiliated organizations, or those of the publisher, the editors and the reviewers. Any product that may be evaluated in this article, or claim that may be made by its manufacturer, is not guaranteed or endorsed by the publisher.
