# Supplementary material for: Identification and analysis of novel endometriosis biomarkers via integrative bioinformatics
Source: Front Endocrinol (Lausanne). 2022 Oct 20;13:942368. doi: 10.3389/fendo.2022.942368 (PMC9630743; doi:10.3389/fendo.2022.942368)

Supplementary Material

**Supplementary Table 1. Information for healthy volunteers and endometriosis patients.**

|  | Normal | Endometriosis | P value |
| --- | --- | --- | --- |
| Number | 10 | 32 | - |
| Age (years) | 30.3 ± 4.9 | 33.33 ± 7.53 | 0.16 |
| BMI | 22.06 ± 2.06 | 21.51 ± 3.52 | 0.56 |
| Stage | N.A. | 2.97 ± 0.92 | - |

**Supplementary Table 2.** **Expression levels of 17 common DEGs in GSE135485.**

| Gene name | Normal  (*n* = 4) | Endometriosis  (*n* = 53) | Log2FC | *p*-value  (*t*-test) |
| --- | --- | --- | --- | --- |
| FABP4 | 0.75 | 138.46 | 7.53 | 0.15 |
| C7 | 44.00 | 7319.93 | 7.38 | 0.00 |
| PTGIS | 53.50 | 1268.74 | 4.57 | 0.00 |
| CFH | 150.00 | 2941.41 | 4.29 | 0.00 |
| CHL1 | 79.25 | 1431.94 | 4.18 | 0.00 |
| PDLIM3 | 103.50 | 1152.87 | 3.48 | 0.00 |
| IGJ | 56.50 | 507.93 | 3.17 | 0.01 |
| FZD7 | 50.00 | 271.06 | 2.44 | 0.00 |
| WISP2 | 39.75 | 169.02 | 2.09 | 0.00 |
| LY96 | 20.25 | 78.87 | 1.96 | 0.04 |
| DEFB1 | 85.00 | 132.43 | 0.64 | 0.17 |
| AGR2 | 372.75 | 488.41 | 0.39 | 0.16 |
| CCL2 | 200.25 | 205.28 | 0.04 | 0.06 |
| PPM1H | 1436.75 | 1115.15 | -0.37 | 0.37 |
| GALNT4 | 125.50 | 74.20 | -0.76 | 0.02 |
| CLDN10 | 287.50 | 152.26 | -0.92 | 0.00 |
| CLDN3 | 406.00 | 44.83 | -3.18 | 0.02 |

Red and blue characters indicate significant enrichment in endometriotic tissues and normal tissues, respectively.

**Supplementary Table 3.** **Expression levels of 17 common DEGs in GSE25628.**

| Gene name | Normal  (*n* = 6) | Endometriosis  (*n* = 7) | Log2FC | *p*-value  (*t*-test) |
| --- | --- | --- | --- | --- |
| PTGIS | 5.32 | 10.73 | 5.41 | 0.00 |
| C7 | 5.97 | 10.50 | 4.54 | 0.00 |
| WISP2 | 4.84 | 8.95 | 4.11 | 0.00 |
| FZD7 | 5.22 | 9.11 | 3.89 | 0.00 |
| PDLIM3 | 5.51 | 9.23 | 3.72 | 0.00 |
| FABP4 | 5.86 | 8.48 | 2.62 | 0.02 |
| CFH | 6.56 | 8.76 | 2.20 | 0.01 |
| LY96 | 6.36 | 7.73 | 1.37 | 0.02 |
| CCL2 | 6.88 | 7.85 | 0.96 | 0.22 |
| IGJ | 5.74 | 6.35 | 0.61 | 0.64 |
| DEFB1 | 8.80 | 8.80 | 0.00 | 1.00 |
| CHL1 | 5.72 | 5.53 | -0.19 | 0.88 |
| CLDN3 | 9.21 | 8.81 | -0.40 | 0.46 |
| CLDN10 | 9.94 | 9.08 | -0.86 | 0.30 |
| GALNT4 | 6.20 | 5.06 | -1.14 | 0.04 |
| PPM1H | 8.54 | 6.78 | -1.75 | 0.00 |
| AGR2 | 11.28 | 9.01 | -2.27 | 0.02 |

Red and blue characters indicate significant enrichment in endometriotic tissues and normal tissues, respectively.


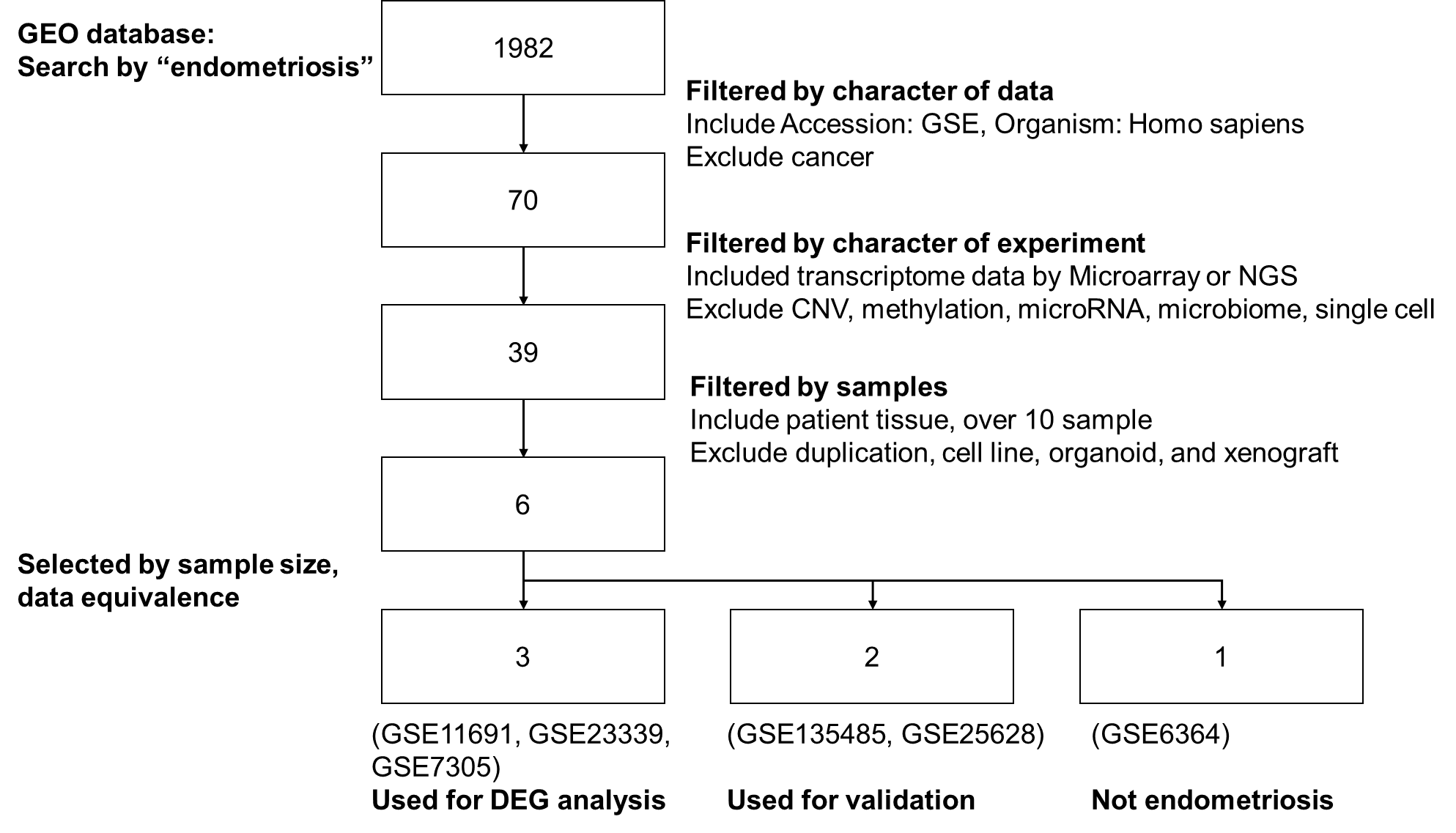


**Supplementary Figure 1. Inclusion and exclusion criteria for selection of datasets from GEO database.** The datasets were obtained by searching the GEO database and filtered by three different criteria. Among six GSE datasets, three gene sets were used for DEG analysis and two were used for validation. One dataset was not from the endometriosis sample but from the normal endometrial tissues.


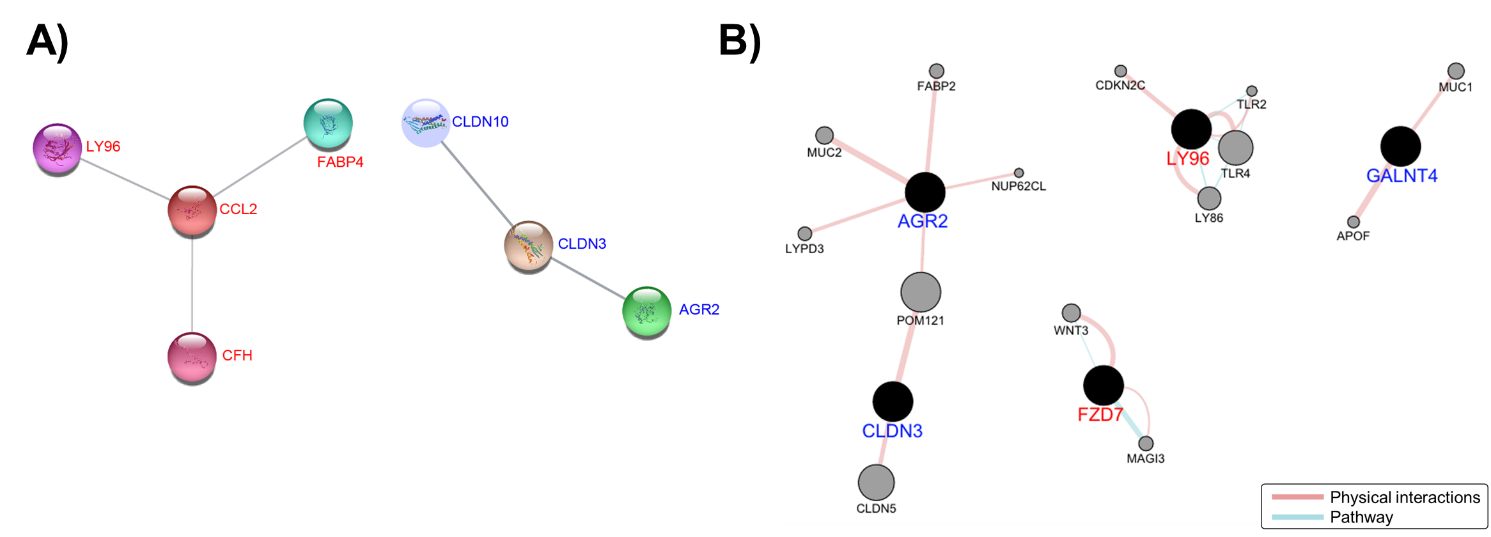


**Supplementary Figure 2. Analysis on the PPI networks of 17 common DEGs in endometriosis.** **(A)** STRING and **(B)** GeneMANIA for Cytoscape plugins were applied to analyze the PPI networks of DEGs from three datasets with log2|FC| > 2. Red character indicates the genes enriched in endometriosis and blue character presents enriched in normal.

**
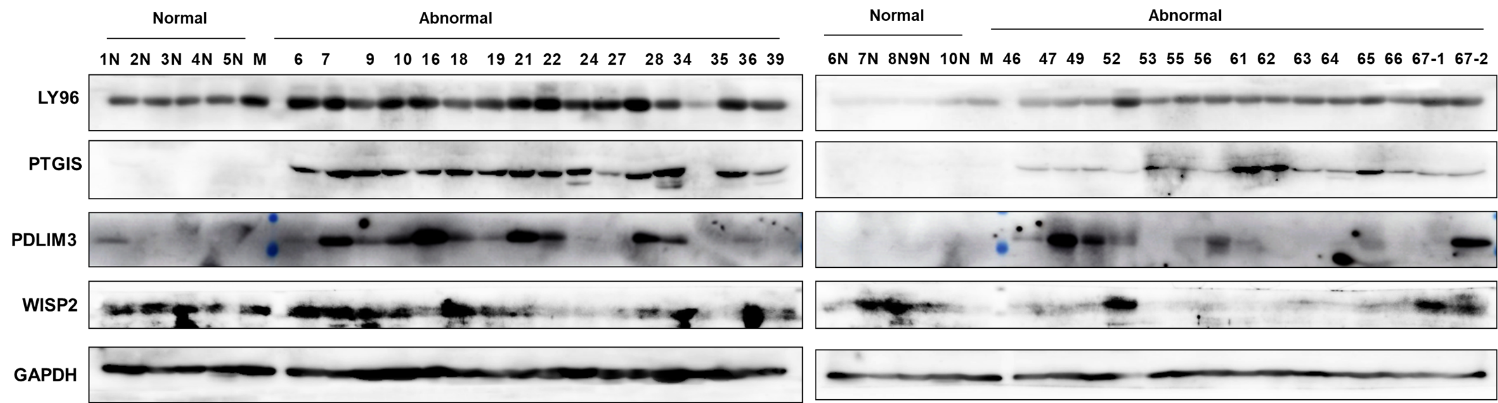
**

**Supplementary Figure 3. The protein expression of LY96, PDLIM3, PTGIS, and WISP2 in endometriosis tissue.** The tissues were homogenized, and total proteins were extracted. The protein expressions of LY96, PDLIM3, PTGIS, and WISP2 were measured by western blot analysis. The expression of GAPDH was used as internal control. N means normal sample and M indicates marker for protein size.

# Supplementary Figure 4. The whole figures of western blot analysis used in this study. All blots were used in Supplementary Figure 3.

#
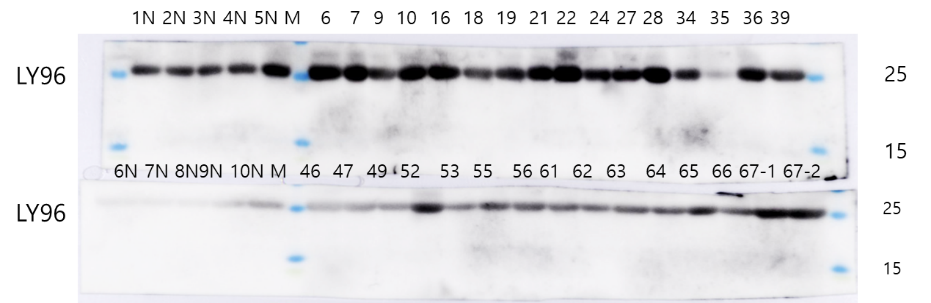


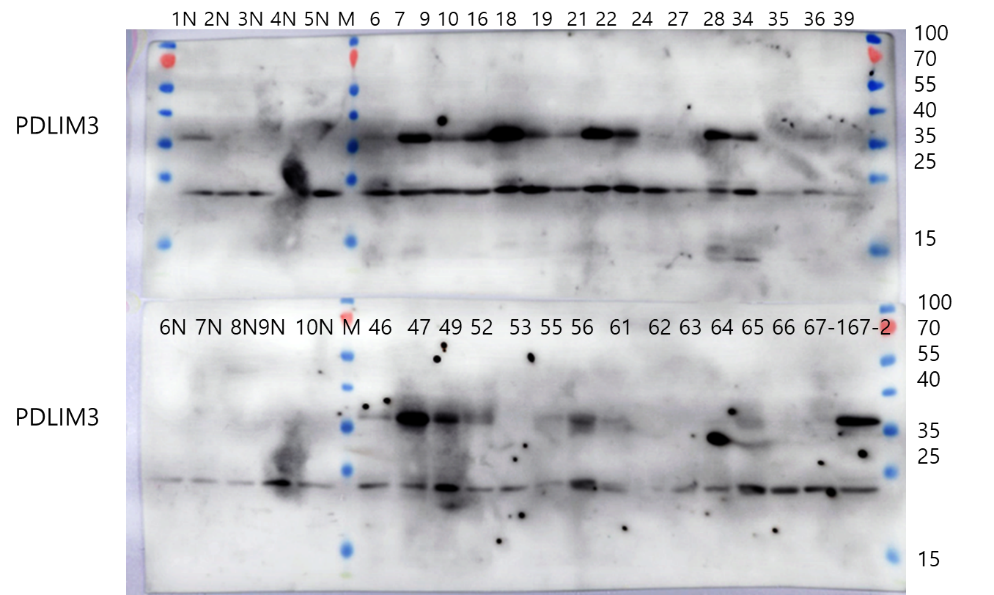


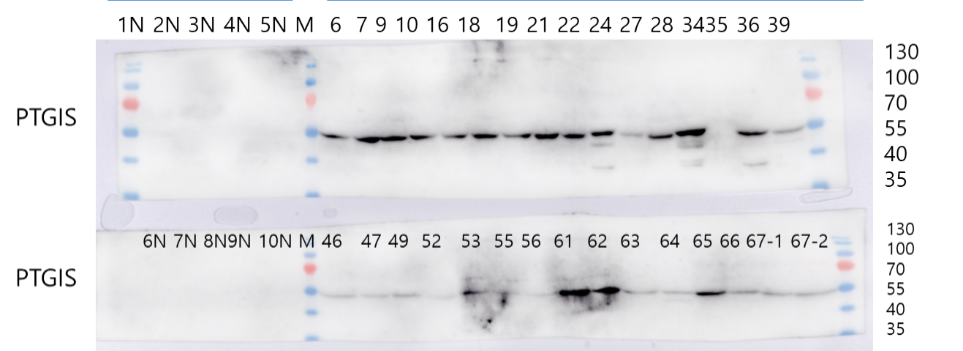


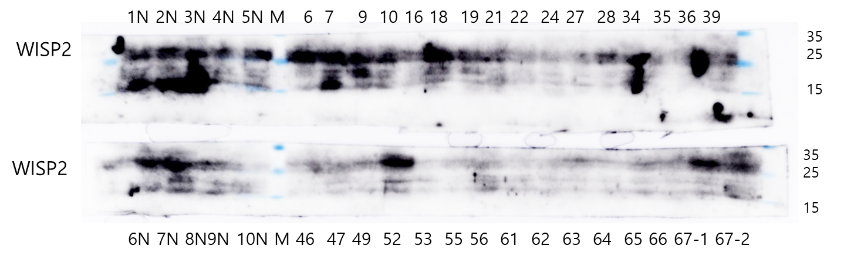


#
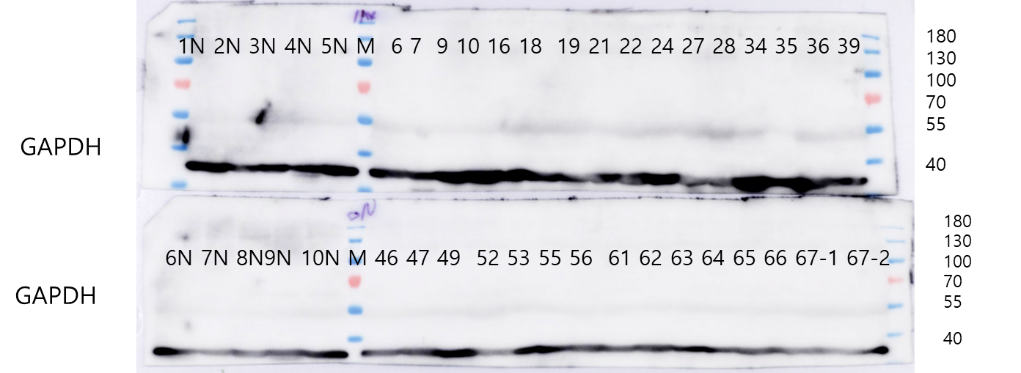

Supplement: Supplementary file 1 [file DataSheet_1.docx]
